# Advances in Rodent Experimental Models of Sepsis

**DOI:** 10.3390/ijms24119578

**Published:** 2023-05-31

**Authors:** Lun Cai, Elizabeth Rodgers, Nick Schoenmann, Raghavan Pillai Raju

**Affiliations:** 1 Department of Pharmacology and Toxicology, Medical College of Georgia, Augusta University, Augusta, GA 30912, USA; 2 Department of Emergency Medicine, Medical College of Georgia, Augusta University, Augusta, GA 30912, USA

**Keywords:** sepsis, shock, cecal ligation and puncture, humanized mice, dirty mice, animal models

## Abstract

In the development of therapeutic strategies for human diseases, preclinical experimental models have a key role. However, the preclinical immunomodulatory therapies developed using rodent sepsis were not successful in human clinical trials. Sepsis is characterized by a dysregulated inflammation and redox imbalance triggered by infection. Human sepsis is simulated in experimental models using methods that trigger inflammation or infection in the host animals, most often mice or rats. It remains unknown whether the characteristics of the host species, the methods used to induce sepsis, or the molecular processes focused upon need to be revisited in the development of treatment methods that will succeed in human clinical trials. Our goal in this review is to provide a survey of existing experimental models of sepsis, including the use of humanized mice and dirty mice, and to show how these models reflect the clinical course of sepsis. We will discuss the strengths and limitations of these models and present recent advances in this subject area. We maintain that rodent models continue to have an irreplaceable role in studies toward discovering treatment methods for human sepsis.

## 1. Introduction

Sepsis affects more than 48 million people annually, leading to the death of approximately 11 million people across the world [1]. Sepsis is a life-threatening clinical condition that may lead to multiple organ dysfunction resulting from a dysregulated inflammatory response to infection [2,3,4]. Sepsis is one of the most common causes of death in intensive care units worldwide [5,6]. The initial symptoms of sepsis include an early hyperinflammatory phase followed by a sustained immunosuppressive phase, referred to as immunoparalysis. The condition is characterized by an intravascular activation of the host’s inflammatory system by which potent mediators of inflammation are released into the circulation [7]. Despite considerable advances in biomedical research, sepsis and septic shock remain a major global health problem [2,8]. Treatment strategies developed in rodent models were not entirely successful in humans, and a viable alternative to animal models is still not available. A deeper understanding of the different experimental models of sepsis, modification of the existing models based on the current knowledge, and a change in approach to study molecular targets could accelerate the discovery of novel treatment strategies, rather than ignoring the use of preclinical models. This review aims to describe the various animal models of sepsis and advances in this area with an emphasis on their methodology, strengths, and limitations.

**Figure 1:**
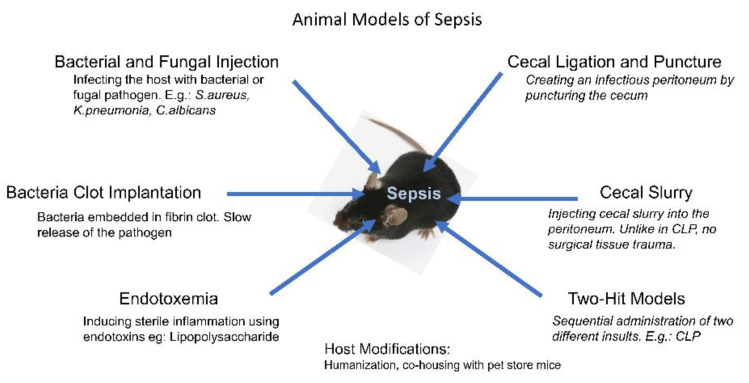
Methods to develop sepsis models. Emerging studies show that modification of the host can reduce the gap between murine models and human sepsis.

During the past several decades, many experimental models of sepsis, such as those using mice and rats, were being used in a number of laboratories. These models are characterized by inflammation, altered cardiac output, mean arterial pressure (MAP), and systemic vascular resistance, with many of them mimicking aspects of human sepsis [9,10,11,12], though all of them have drawbacks. These studies demonstrate that the early physiological and molecular processes in sepsis can be explained using animal models. The methodologies to develop these models (Table 1) include, but are not limited to, the exogenous administration of a toxin such as a lipopolysaccharide (LPS), administering a pathogen, or enabling the release of endogenous pathogens [10]. The advent of genetically modified mouse strains advanced our understanding of the role of specific genes in isolation in murine sepsis. Modifying the host animals by humanizing the immune system and making the dirty mouse by changing the housing environment are two strategies that have been employed to modify the host to make it closer to a human system [13]. These investigations allowed the identification of new biomarkers, drug targets, and critical signaling pathways that regulate outcome following sepsis [14,15,16,17]. A number of biomarkers have been identified for sepsis, which include: cytokines, chemokines, pattern recognition molecules, miRNAs, and metabolites [16,18]. Furthermore, there have been attempts to differentiate sepsis from systemic inflammatory response using biomarkers [16]. It is more likely that a combination of biomarkers is necessary in the diagnosis and treatment of sepsis [19]. These biomarkers are well described in a recent review article [16,18,19,20]. In this review, we will discuss the various sepsis models developed in the rodents (Figure 1), including bacterial and fungal infection models, bacteria clot implantation models, endotoxemia models, intraperitoneal sepsis models, and two-hit models; the relevance of genetic background and phylogenetic distance; and humanized mice and “dirty” mice in sepsis studies.

## 2. Bacterial and Fungal Infection Models

Several laboratories have used bacterial species such as *Escherichia coli*, *Pseudomonas aeruginosa*, *Staphylococcus aureus*, *Klebsiella pneumoniae*, *Streptococcus pneumoniae*, and *Streptococcus pyogenes* to induce sepsis in animal models [21,22,23,24,25]. While *E. coli* and *Pseudomonas aeruginosa* cause Gram-negative sepsis, *Staphylococcus aureus* and *Streptococcus pneumoniae* are Gram-positive pathogens. Inducing sepsis by injecting individual pathogens represents infections caused in patients by a single pathogen [26]. Although these models exhibit a similar cytokine response as seen with the endotoxin model [27], the route of administration has a bearing on the magnitude of the host response observed in these models with the intravenous route eliciting a stronger inflammatory response than other routes [28]. Due to the direct and immediate effects on endothelial cells that line the vasculature, intravenous injection produces a strong and quick proinflammatory immune response that may in effect be stronger than the typical host response to infection. Compared with single-bolus injection in the rat, higher doses of *E. coli* administered over several hours have often produced a biphasic response, with an early rise and late fall in cardiac output [29,30]. The pathogen infection model allows the study of antimicrobial host responses, including clearance of the pathogen, and the progression of infection in relation to disease severity [26].

Sepsis may also be induced in experimental animals by intranasal infection using the common Gram-negative and Gram-positive community-acquired pneumonia-causing pathogens, *Klebsiella pneumoniae* or *Streptococcus pneumoniae* [21,31]. The intranasal administration causes a localized early inflammatory response due to pulmonary inflammation along with a strong cytokine response and the homing of neutrophils and macrophages into the inflamed lungs [26]. The bacteria may also be injected directly through the trachea or nasal passage of the animals, eliciting a pathogen-dependent response. *Pseudomonas aeruginosa* requires large doses and produces an acute response leading to rapid pneumonia within 24 h. Infection with *Klebsiella pneumoniae* or *Streptococcus pneumoniae* produces a steadily increasing bacterial load resulting in a system-wide infection. An association of pneumonia-induced mortality following intranasal infection with age remains debatable [32,33]. One study showed an increased mortality in the young compared with the old following inoculation with *Francisella novicida* [34]. A different study found increased expression of bacterial ligands in the lungs after intranasal infection with the pneumococcus and a positive correlation with increased susceptibility to *pneumococcal pneumonia* in aged mice, with concomitantly enhanced cellular senescence [35]. When aged and young rats were compared after inoculation with *L. monocytogenes*, Antonini et al. found altered lung defenses in the aged rats making them more susceptible to lung injury and inflammation [36]. It is also suggested that, as there is an increase in mortality from sepsis itself due to aging, “a certain aspect of sepsis pathophysiology that appears to be enhanced in an age-associated fashion may not be a direct age-dependent phenomenon but rather a reflection of increased severity of disease” [37].

Apart from the models developed with various bacterial strains, several fungal models of sepsis also exist. Although Candida is a commensal organism in 30–60% of healthy individuals, it can induce sepsis in humans by disrupting the intestinal mucus barrier following injury [38]. In intensive care units, candidemia is reported as the fourth most common bloodstream infection [39]. When the fungus is introduced in systemic circulation, mice have been shown to develop kidney failure and septic shock reflective of human sepsis [40]. Others have shown that mice infected with live *C. albicans* develop clotting disorders such as decreased clotting times and thrombocytopenia [41]. In another study, *Candida auris* bloodstream infection was shown to induce upregulation of the PD-1/PD-L1 immune checkpoint pathway in a mouse model [42]. The authors suggested that this model could provide an easy preclinical platform to investigate immune checkpoint blockade and combination therapy with antifungals [42]. The potential of introducing individual pathogens of choice to animals provides the benefit of understanding and determining the mechanisms relating to individual bacterial strains [24].

## 3. Bacteria Clot Implantation Models

The implantation of specific amounts of bacteria embedded in fibrin clots into the abdominal cavity allows the slow release of bacteria and has been used to study early and late phases of sepsis in small and large animal models [43,44]. It is claimed that this model produces a progressive septic process that closely mimics human sepsis [45]. When Ahrenholz et al. administered *E. coli*-impregnated fibrin clots into the rat peritoneal cavity, 24 h mortality was abolished (from 100% to 0%) as compared with the bacteria alone [46]. However, the 10-day mortality rate was 90%, with 100% developing intraperitoneal abscesses. In a canine sepsis model developed by implanting an *E. coli*-infected clot into the peritoneum of conscious unsedated dogs, septic shock produced a profound decrease in systolic ventricular performance associated with ventricular dilatation [47]. This study also found a significant shift in the diastolic volume/pressure relationship during septic shock [47]. These models replaced fecal pellet models that were initially formulated to separate the development of peritonitis from abscess formation [48]. In a bacterial clot model, the advantage is that the chosen bacteria can be inoculated with the fibrin clot to slow the release and the animals’ manifest metabolic and inflammatory dysregulation for a prolonged period of time. While the use of a single bacterial strain is useful in understanding its role in sepsis pathogenesis, this may not adequately reflect the physiological alterations seen in polymicrobial sepsis.

## 4. Endotoxemia Models

One of the models of systemic inflammatory response syndrome (SIRS) that imitate the inflammatory reaction in sepsis is developed by the administration of bacterial- or fungal-derived pathogen-associated molecular patterns (PAMPs). This may be accomplished by intravenous, intraperitoneal, or intranasal/intratracheal processes [29]. Lipopolysaccharide (LPS) from Gram-negative *Escherichia coli* or *Klebsiella* is commonly used to elicit SIRS. LPS, termed “endotoxin” due to the toxicity observed for many Gram-negative organisms, binds to the toll-like receptor (TLR4) on host cells and activates downstream inflammatory pathways [49]. This method involves the induction of a robust and immediate inflammatory response that is similar to the activation of the innate immune system seen in human sepsis. Some of the advantages of the endotoxemia method are its ease of use and the reproducibility of the results. However, while using LPS is representative of Gram-negative bacteria, the method does not account for the effect of Gram-positive and polymicrobial sepsis. It lacks the infectious component found in human sepsis.

Experimental animals exhibit a dose-dependent response to LPS [27,29,32]. Large doses of LPS may resemble a small subset of human sepsis patients that are reminiscent of certain very fulminant forms of Gram-negative bacterial infection in humans, for example, meningococcal sepsis or toxic shock syndrome [50]. To mitigate the limitations of a single dose, continuous infusions of low-dose LPS have been used to create a hyperkinetic state as a model for severe infection [51]. The LPS model failed to replicate some important aspects of the immune response, such as the cytokine-mediated hyperinflammatory phase with the following immune-suppressive phase [52]. It did, however, reproduce the hyperdynamic cardiovascular state.

The cell surface M1 protein of Gram-positive *Streptococcus pyogenes* is another PAMP that stimulates the immune system to imitate the sepsis-induced inflammatory response [53]. Neutrophil-dependent organ damage independent of platelets can be caused by M1 challenge and increases the production of cytokines, chemokines, and tissue factors [54]. Further, the M1 protein was found to contribute to the development of septic shock through impairment of the contractility of the vascular wall [55]. An intraperitoneal challenge of mice with the yeast-derived cell surface polysaccharide zymosan A (e.g., from *Saccharomyces cerevisiae*) led to the activation of TLR2 and elicited a powerful inflammatory response with marked biosynthesis of eicosanoids and influx of neutrophils [56,57]. Rao et al. reported that the neutrophil influx was regulated by lipoxigenase and cycloxygenase metabolites [56]. Zymosan A activates a triphasic immune response similar to chronic sepsis in human patients [26]. First, there is a substantial inflammatory response with elevated levels of TNF-α, and IL-6, with acute peritonitis followed by significant mortality. The second phase consists of chronic low-grade inflammation that later progresses to a final stage characterized by generalized inflammation, tissue injury, organ failure, and death [53,58]. This model appears to have a few similarities to human sepsis, such as prolonged disease and multiorgan failure occurring biphasically [57]. Nevertheless, SIRS as a descriptor of sepsis pathobiology is being challenged [2].

## 5. Intraperitoneal Sepsis

The intraperitoneal sepsis model is considered closest to human sepsis. The three commonly used models of intraperitoneal sepsis are cecal ligation and puncture (CLP), cecal slurry (CS), and colon ascendens stent peritonitis (CASP) [53].

Cecal ligation and puncture: CLP is one of the regularly used and well-accepted sepsis models, and it is still considered a gold standard because it has many similarities to human sepsis. These similarities include a decrease in white blood cells, low platelet count, hypotension, elevated levels of proinflammatory cytokines, and markers of reduced organ function [59,60,61,62]. CLP is induced by a simple surgical procedure beginning with a midline incision to open the peritoneal cavity [62,63,64]. The cecum is exposed, ligated below the ileocecal valve, and punctured across the cecum using a sterile needle. A small amount of fecal matter is gently squeezed out through the puncture site. The cecum is placed back into the peritoneal cavity and the abdominal wall closed. As a control, a sham group can be maintained with the laparotomy and manipulation of the bowel, without performing ligation and perforation of the cecum. Following the procedures, the mice are resuscitated using normal saline [59]. The goal in this technique is to perforate the cecal barrier leading to peritoneal infection with the fecal content. Some investigators remove the infectious cecal remnant and clean the peritoneum after 24 h for a more controlled maintenance of systemic infection. By varying the number of cecal punctures and the size of the needle used, fecal spillage can be altered to modulate the severity of sepsis. Nevertheless, there are several limitations of the model, including the difficulty in controlling cecal content release as well as inter- and intra-experimental variability in severity and mortality. Furthermore, several therapies developed based on the promising results obtained using this method could not be translated to the clinic [59].

There are major differences between the widely used LPS method and the CLP method in simulating human sepsis in experimental models. While LPS is a well-defined PAMP and triggers systemic inflammation, the CLP model is polymicrobial in origin and exhibits more characteristics of human sepsis, such as an infectious component, microvascular dysfunction, and temporal changes in physiological parameters [65]. Inflammatory cytokine responses to CLP develop more slowly than the LPS challenge, even at similar illness and death rates [66], possibly due to a more protracted disease process with infected foci.

The cecal slurry (CS) model is another intra-abdominal sepsis model used by some investigators. This method involves the intraperitoneal injection of a specific amount of cecal content, resulting in a stronger but shorter early inflammatory response than CLP [32,67,68,69]. When rats were injected with cecal slurry into the peritoneum, a reduced oxygen delivery with decreased tissue PO2 was observed at 6 h, whereas by 24 h myocardial and circulatory function were largely recovered [70]. Nevertheless, the authors noted a worsening clinical severity and increasing biochemical dysfunction.

The major difference between the CLP and CS model is the lack of surgical tissue trauma and ischemic tissue in CS that results from standard CLP methods. Another advantage to CS is that this method can be performed on neonatal mice. Wynn et al. showed that neonatal mice were more susceptible to sepsis and mounted a markedly attenuated systemic inflammatory response compared with young adult animals when sepsis was induced with CS [71]. Their small size and risk for maternal cannibalization post-procedure are two of the challenges of performing the CLP procedure in neonatal animals [71]. A major advantage of the CS model is its reproducibility between experiments, which can be attributed to the more controlled infectious source used. However, different batches of the preparation can produce varying results. It was found that CS prepared in 15% glycerol–PBS and stored at −80° maintained bacterial viability for at least 6 months [69]. The authors observed age- and dose-dependent mortality with high reproducibility, with mortality correlating strongly with the circulating bacterial levels, suggesting an infection-mediated death [69].

Colon ascendens stent peritonitis (CASP) is a relatively new method used to produce murine abdominal sepsis [72]. To induce CASP, a small stent is inserted into the ascending colon after a midline laparotomy, which allows a constant flow of fecal matter from the colon into the abdominal cavity [72,73]. The CASP model reproduces the organ dysfunction seen in human sepsis, specifically the changes in lung, kidney, and bone marrow function [10,73,74,75,76,77,78]. The CASP procedure results in peritonitis, systemic bacteremia, organ infection by gut bacteria, and elevated pro- and anti-inflammatory cytokines [79]. The degree of the septic insult can be controlled by changing the size of the stent catheter [76,77]. This procedure has been successfully performed in both rats and mice [80]. Using gene knockout models, Zantl et al. reported that CASP sepsis survivability is dependent on interferon gamma but independent of TNF-α [72]. When CASP and CLP models were compared, while the bacterial load and inflammatory markers steadily increased within 24 h following CASP, the CLP mice exhibited reduced levels of bacteria and serum cytokines [73]. Recently, Ai et al. observed a full range of polymicrobial (viral, bacterial, and parasitic) agents following CASP surgery in a mouse [81]. Another study recently showed that the CASP model is a superior animal model to study disseminated intravascular coagulation (DIC) in murine abdominal sepsis [82].

## 6. The Two-Hit Models

The two-hit model was developed to mimic biphasic multiple organ failure (MOF). The initial insult primes the host, whereas the subsequent event leads to further immune activation and MOF [83]. Essentially, it is assumed that the host becomes more susceptible to a secondary insult after the primary event. In a recent study, to test the effect of resolvin D2 at different stages of sepsis, a two-hit model was employed—an initial CLP surgery followed by a secondary lung infection with Pseudomonas aeruginosa—and it was found that resolvin D2 promoted mechanisms of host defense [84]. To establish a murine model of nonpulmonary sepsis, researchers used CS as the initial insult followed by hyperoxia [85]. In another study, mice subjected to CLP surgery coupled with a secondary *P*. *aeurginosa* treatment showed increased susceptibility to infection after the initial sepsis event [86]. There is no well-defined sequence or nature of individual hits, as each insult is chosen to suit the question addressed. One such model involved an initial event of hemorrhagic shock followed by endotoxin treatment in a rabbit model [87], whereas in another model the superior mesenteric artery was clamped to induce an ischemia/reperfusion effect with later administration of endotoxin [88]. In another two-hit model, sepsis was induced in the mice by CLP after a focused blast wave applied lung contusion to test the effect of neutrophil depletion on lung injury [89]. Some investigators use multi-hit models [12]. However, as it was pointed out, despite these efforts and novel methodologies, murine research in trauma and sepsis is still evolving and can be improved [90].

## 7. Genetic Background and Phylogenetic Distance

Laboratory mice and rats have been very useful to study the role of individual genes or molecular pathways in biological processes. Although studies using congenic mice are ideal to understand mechanistic aspects in a reductionistic manner, the influence of background genes on such mechanistic pathways have been seldom investigated. This is one of the major drawbacks of using animal models. The response to sepsis induction in any animal may depend on the genetic makeup of the animal. For example, different inbred congenic mouse strains may display a divergent immune response due to genetic polymorphisms and mutations [91]. A poor survival rate was observed in C57BL/6J mice, that had lower IFN-γ serum levels, compared with CD1 mice when challenged with zymosan [92]. The host genetics is also an important factor to consider in developing rodent models of virus-induced sepsis. This barrier may be overcome by creating a humanized immune system in the mouse or developing transgenic animals that express human receptors for viruses. An example is the development of the mouse model for SARS-CoV-2 infection. SARS-CoV-2 fails to infect the wild-type mouse because mouse ACE2, as opposed to hamster ACE2, has different amino acids in the spike-binding domain compared with human ACE2 [93]. K18-hACE2 transgenic mice generated with the altered ACE2 developed severe disease in the lung when infected with SARS-CoV-2 [94]. Furthermore, SARS-CoV-2 with a modified receptor-binding domain was able to infect both mice and rats [95]. The overwhelming viral infection that the host response fails to clear results in severe sepsis or death [13].

Furthermore, the dependence of the sepsis phenotype on genetic makeup may also suggest a multigenic influence on sepsis susceptibility and outcome [96]. The authors studied 2261 patients with sepsis and 13,068 controls and found a polygenic association involving hundreds of genetic variants [96]. When the expression of genes in the peritoneal cells of mice challenged with LPS was compared with that of LPS-treated human monocytes, a significant overlap of differentially expressed genes was found, indicating a common molecular response in the mouse and the human despite the phylogenic difference [97]. This may not be surprising considering the high degree of similarity between the mouse and human genomes [98]. However, the extent of similarity in gene makeup required in the mouse to simulate human sepsis is unclear. The failure of drugs developed in preclinical models in the treatment of sepsis is at least partially due to this disparity and has accelerated the search for alternative strategies to develop models closer to human sepsis.

## 8. Humanized Mice and “Dirty” Mice in Sepsis Studies

One emerging line of investigation uses humanized mice [13,99]. Most of the humanized models use immunodeficient mice reconstituted with specific human immune compartments with the belief that the replacement of the mouse immune system with a human immune system is key to finding commonality between the mouse and the human. While the severe combined immunodeficient (SCID) mouse is a commonly used model, the discovery that IL-2Rγ^null^ on Rag2^null^ had better engraftment of human cells led to the development of a series of genetically modified strains of SCID and Rag1/2^null^ mice with the IL-2Rγ^null^ mutant gene [100]. The studies utilizing humanized mice to understand sepsis began with the report from Hotchkiss and colleagues using NOD-*scid* IL2rγ^null^ mice transplanted with hCD34^+^ hematopoietic cord blood stem cells [101]. Sepsis was induced in these mice by CLP at 8 weeks after the xenotransplant, and the model recapitulated many of the classic findings in patients with sepsis. An exhaustive review of these studies can be found in a recent review by Ludanski [13]. The author also lists a number of limitations of this approach, including the extent of reconstitution of the human immune system attained in the mouse.

While the use of congenic animals may be substituted by outbred animals to mimic heterogeneity in human genetics, laboratory animals pose additional questions with regard to the influence of the controlled environment in which these animals are raised. The controlled laboratory housing can potentially skew the immune system. It is well known that mice that are bred and raised in pathogen-free colonies demonstrate a different level of susceptibility to immune-mediated diseases and altered physiology when compared with mice housed in conventional colonies [102,103,104]. Recently, Beura et al. reported that the housing environment has a profound influence on the immune system of mice and less clean environments result in immune systems closer to those in humans [105]. Importantly, they found that the immune system of the laboratory mice were more like that of newborn humans, with a deficiency in effector-differentiated and mucosally distributed memory T cells, while these immune cell populations were found in pet store mice living in dirtier environments [105]. When the laboratory mouse environment was made similar to that of the pet store mice, the gene expression of the blood cells from these mice displayed more similarity to immune signatures of adult humans. When sepsis was induced by CLP in mice housed in a pathogen-free facility and laboratory mice co-housed with pet store mice (dirty mouse), the dirty mice exhibited more severe weight loss and increased acute mortality [106]. According to the authors, similar results were obtained in CS-induced sepsis and LPS endotoxemia models. The development and characterization of the dirty mouse model was another milestone in experimental sepsis research; however, its reproducibility across laboratories remains to be established due to the lack of a well-defined “dirty” environment.

## 9. Conclusions

Animal models have been fundamental to the understanding of human health and development of therapies for several human diseases, though currently there is a high attrition rate in animal-to-human translation [107,108]. The experimental models of sepsis vary in their methods and species used, though mice and rats have been the most preferred species. Among the methods employed, the CLP and CS methods are both simple and reflective of polymicrobial sepsis, while CASP is a more complex procedure. However, the translational failure of therapeutics successfully tested in animal models prompts the modification of existing sepsis models or discovery of newer models. Recent developments have been in two major areas: (1) the development of humanized mice and (2) the use of dirty mice in sepsis research. The advent of humanized mouse models, generated by transplanting the human immune system into immunodeficient mice, hold promise in narrowing the gap between the mouse models and human sepsis as the host mice carry human myeloid and lymphoid cells [100]. The other emerging concept that laboratory mice are bred in a cleaner environment and therefore their immune constitution might be different from that of humans also has great potential in sepsis investigations. The introduction of dirty mice to sepsis research is promising; however, additional studies are needed to better characterize this mouse model. Furthermore, the generation of dirty mice by co-housing them with pet store mice poses several challenges [109]. There will be several limitations to sepsis research if studies are restricted to human sepsis. While some correlative studies can be performed in human sepsis, the initial testing of pharmacological agents and determination of the mechanistic basis of their action are not possible with human subjects. Therefore, studies in preclinical models are necessary. It is unlikely that an in silico model that takes the place of a preclinical sepsis model will be available soon. Given the limitations, it is necessary to continue preclinical studies using appropriate small animal models, but the investigational approach may need to be reevaluated. While most of the studies in translational sepsis research have focused on immune-modulatory pathways, a fresh look at other molecular mechanisms, such as in metabolic and bioenergetic processes, may lead us to more promising targets in sepsis.

## Figures and Tables

**Table 1:** Rodent Models of Sepsis.

	Pros	Cons
Bacterial and Fungal Infection	Selected pathogens of choice can be tested, lack of surgical insult, and ability to study progression of infection in relation to severity.	Strain, dose, and route dependence on severity; single pathogen may not reflect human sepsis.
Bacteria Clot Implantation	Allows slow pathogen release, produces progressive sepsis, and prolonged immunometabolic dysregulation.	Reproducibility depends on clot standardization.
Endotoxemia	Simple procedure, reproducibility, and acute response.	Dependence on toxin, dose, and route. Differs from clinical sepsis.
Intraperitoneal		
Cecal Ligation and Puncture	Polymicrobial sepsis. Cardio-metabolic and immune response similar to clinical sepsis. Organ dysfunction. Simple surgical procedure.	Variability of the model (needle size, number of punctures, ligated cecum length). Surgical insult.
Cecal Slurry	Reproducibility, ease of use, lack of surgical trauma, and organ dysfunction.	Batch-to-batch variation in the slurry.
Colon Ascendens Stent Peritonitis	Polymicrobial infection, organ dysfunction, and inflammatory response.	Surgical insult, variability due to stent size, and challenging surgical model.
Two-Hit (e.g., CLP followed by lung infection)	Mimics biphasic multiorgan failure.	Variability depending on duration between hits, nature of each hit, and sequence of hits.

## Data Availability

Not applicable.
